# The efficacy of taking traditional Chinese medicine orally in renal interstitial fibrosis: A protocol for a systematic review and meta-analysis

**DOI:** 10.1097/MD.0000000000022181

**Published:** 2020-09-18

**Authors:** Guang Yu, Mao Guo, Junju Zou, Xiaotao Zhou, Yuerong Ma

**Affiliations:** a College of Basic Medicine, Chengdu University of Traditional Chinese Medicine, Chengdu; b Pain Clinic, The People's Hospital of Luzhou, Luzhou; c Pathology Department, Affiliated Hospital of Chengdu University of Traditional Chinese Medicine, Chengdu, China.

**Keywords:** meta-analysis, renal interstitial fibrosis, systematic review, traditional Chinese medicine

## Abstract

**Background:**

By now, the incidence of chronic kidney disease (CKD) is increasing. The development of various CKD is attributed to the continuous aggravation of renal interstitial fibrosis (RIF) in the process of end-stage renal disease (ESRD). Oral treatment of traditional Chinese medicine (TCM) is one of the therapies for RIF. Randomized controlled trials (RCTs) of TCM treatment RIF have been reported, but its effectiveness and safety have yet been systematically investigated. Therefore, through the systematic analysis and meta-analysis, our study will summarize the effectiveness and safety of oral treatment RIF of TCM, in order to provides scientific reference for clinical practice.

**Methods:**

This protocol follows Preferred Reporting Items for Systematic Evaluation and Meta-Analysis. RCTs will be only selected. Such databases as the PubMed, China National Knowledge Infrastructure (CNKI), China Science and Technology Journal Database (VIP), Excerpt Medical Database (Embase), WanFan Data, Chinese Biomedical Literature Database (CBM), WHO International Clinical Trials Registry Platform will be searched from the inception to June, 2020 to collect the RCTs about taking TCM orally in treating RIF. The literature according to the inclusion and exclusion criteria, data-extracted and the methodological quality evaluated will be performed independently by 2 reviewers. The clinical outcomes including renal function indices (Scr, BUN, 24-hour urinary protein quantity) and Indicators of RIF (TGF-β1, Notch1, Jagged-1). The risk of bias included in the RCTs will be evaluated by the bias risk assessment tool provided in the Cochrane System Evaluation Manual 5.1.0. Review Manager 5.3 provided by the Cochrane collaboration network will be used to process the data.

**Results and conclusion:**

Some more targeted and practical results about the efficacy of taking TCM orally in RIF have been provided by our study. The available evidence suggests that the therapeutic effects of combining TCM with Western medicine therapies is much better for RIF than Western medicine therapies only.

## 1 Introduction

By now, the incidence of chronic kidney disease (CKD) is increasing, which has become another public health problem that has attracted wide attention all over the world. In America, the prevalence of CKD in adults was 13.2% in 2014, which is expected to rise to 14.4% in 2020 and 16.7% in 2030.^[1]^ In China, the prevalence of CKD in adults was 10.8% in 2012, and the number of CKD patients was about 119 million.^[2]^ Up to 10% to 15% of adults have been affected by CKD in worldwide.^[3]^ CKD has become another threat to human health. Studies have shown that RIF is the common pathway and main pathological basis of kidney disease progression to renal failure, and is the common outcome path of most CKD.^[4,5]^ This process involves the injury of renal tubular epithelium and renal interstitium, the production of cytokines and inflammatory mediators, the infiltration of inflammatory cells, the proliferation of fibroblasts, and the increase of extra cellular matrix (ECM). And the related signal transduction pathway mediated by cytokines plays an important role in this complex process.^[6]^ The main signaling pathways involved include TGF-β-Smad signaling pathway, Wnt/β-catenin signaling pathway, Notch signaling pathway and Hedgehog signaling pathway and so on. The expression of fibrogenic factors in these pathways is closely related to the occurrence and development of RIF.^[7–10]^ Therefore, observing the expression of fibrogenic factors can reflect the state of an illness of RIF to some extent.

There is no disease of RIF in TCM, but according to its pathogenic characteristics and the clinical manifestations of it, it can be classified into the categories of edema, kidney overstrain, retention of urine, etc.^[11]^ TCM believes that the “kidney collaterals” scattered in the kidney can cause obstruction of collaterals and collaterals due to the imbalance of Qi and blood and blood stagnation for a long time. Tianshi Ye who was a Famous doctor in Qing Dynasty believed that it should take “dredging collaterals” as the treatment method for the treatment of collateral disease. And in addition to promoting blood circulation or removing blood stasis, the use of insect drugs were particularly necessary. Therefore, according to the treatment principle of “strengthening the body and removing blood stasis, reducing turbidity and unblocking collaterals”, the treatment of RIF can be achieved through the application of oral TCM.

According to retrospective literature reviews over the years, the therapeutic effects of combining TCM with Western medicine therapies is much better for RIF than Western medicine therapies only.^[12–14]^ However, the exact effect and safety of oral TCM on RIF has not been systematically studied. By systematic evaluation and meta analysis, this study will summarize the efficacy and safety of taking TCM orally in the treatment of RIF, so as to provides scientific reference for clinical practice.

## 2 Research objective

Systematic evaluation and meta analysis aim to systematically assess the efficacy and safety of taking TCM orally in the treatment of RIF.

## 3 Methods

The review protocol has been registered in the International Platform of Registered Systematic Review and Meta-Analysis Protocols (INPLASY). The registration number is INPLASY202080042 (DOI number is 10.37766/inplasy2020.8.0042). Systematic evaluation will be conducted by Cochrane Handbook for Systematic Reviews of Interventions guidelines manual and reported according to the preferred reporting items of the Systematic Review and Meta-Analysis Protocols (PRISMA-P) guidelines. Moral approval or informed consent is not required in this study as it belongs to a secondary study based on certain previously disclosed data.

### 3.1 Dissemination plans

We will disseminate the results of this systematic review by publishing manuscripts in peer-reviewed journals or publishing relevant findings at relevant conferences.

### 3.2 Inclusion and exclusion criteria

#### 3.2.1 Study designs to be included

RCTs of taking TCM orally for RIF in chronic kidney disease. Randomized grouping regardless of whether it is single-blind, double-blind or non-blind.

#### 3.2.2 Research object

Patients with unlimited race, nationality, age, and sex has a history of CKD or systemic disease involving the kidney.

#### 3.2.3 Interventions

The treatment group was treated with integrated Chinese and western medicine (basic Western medicine + TCM) or TCM only. The TCM was administered orally. The course of treatment is unlimited.

#### 3.2.4 Comparator

The control group was treated with western medicine only or blank or placebo.

#### 3.2.5 Study designs to be excluded

Exclude other organ fibrosis diseases that affect fibrogenic factors. Excluding non-randomized controlled trials, non-oral administration, the control measures are TCM, and the control measures are not clearly described. Exclude the incomplete data and similar data.

#### 3.2.6 Type of outcome

##### 3.2.6.1 Main outcome(s)

The efficacy of oral TCM in RIF will be evaluated by serum creatinine concentration (Scr), blood urea nitrogen (BUN), Notch1, Jagged-1 and urine TGF-β1 content and 24-hour urinary protein quantity.

##### 3.2.6.2 Additional outcome (s)

The additional outcomes include the effective rate and adverse reactions.

### 3.3 Information sources and Search strategy

Suitable studies will be systematically searched from electronic databases including PubMed, CNKI, VIP, Embase, WanFan Data, CBM and WHO International Clinical Trials Registry Platform from their inception to June, 2020. Search terms include “renal interstitial fibrosis” or “Renal Fibrosis” [Title /Abstract], “Medicine, Chinese Traditional” or “TCM” [MeSH Terms], “Drugs, Chinese Herbal” or [MeSH Terms], “Random” or “RTCs” [MeSH Terms], Filters: Clinical Trial; Humans, #1 and #2, #1 and #3. Languages are limited to English and Chinese. The strategies will be developed based on the guidance of the Cochrane Handbook. Table 1 shows PubMed's search strategy. Similar strategies will be modified and applied to other electronic databases.

**Table 1:**
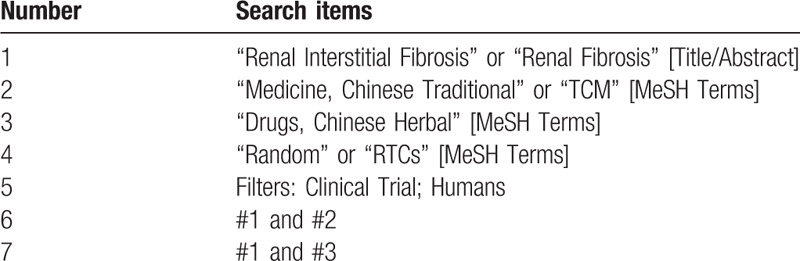
Search strategy for PubMed.

### 3.4 Literature screening and management

**Figure 1:**
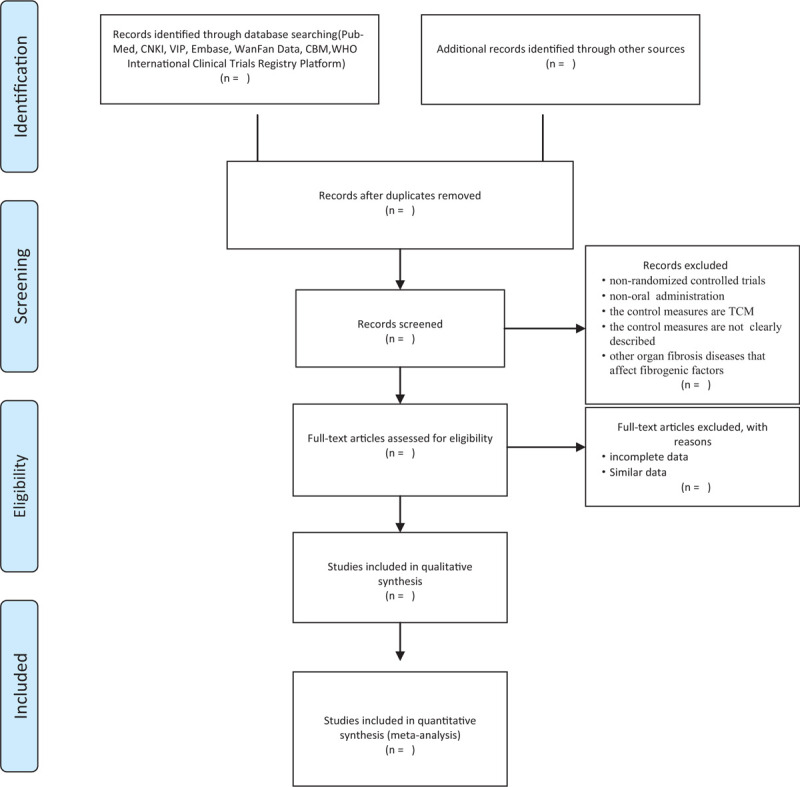
Flow diagram of literature search.

Two researchers will carry on the literature retrieval independently and use the Noteexpress software to conduct a preliminary review of the literature, then read the title and abstract of the article for a preliminary screening, and then exclude it by reading the full text. If there are some differences on inclusion and exclusion, we will have a panel discussion. Details of the entire selection process will be shown in the PRISMA flow chart (Fig. 1).

### 3.5 Data extraction and management

According to the evaluation manual of the Cochrane intervention system, 2 researchers will independently extract the data of the research that meet the inclusion criteria, and then check the extracted contents one by one. In case of disagreement, it is solved by research the literature and discussion. If the research report is still not available, remove the document. The extracted contents mainly include the basic situation, sample size, design method, intervention measures, intervention time, curative effect index and measurement value of the included study.

### 3.6 Dealing with the missing data

The missing data will affect the results to some extent. When we make an exclusion decision, we need to contact the authors to request missing or incomplete data for further checking and recording. If there is no exit or acquisition of relevant data, we will exclude them from the analysis.

### 3.7 Quality evaluation

The risk of bias included in the RCTs will be evaluated by the bias risk assessment tool provided in the Cochrane System Evaluation Manual 5.1.0,^[15]^ including the generation of random sequence schemes, whether to use allocation hiding, whether to implement blind methods, whether the result data is complete, whether to selectively report results, and other sources of bias. The above 6 items will be evaluated with “yes” (low bias), “no” (high bias) or “unclear” (lack of relevant information or uncertainty of bias).

### 3.8 Statistical methods and heterogeneity assessment

The Revman 5.3 software provided by the Cochrane collaboration network is used for data processing. The measurement data are expressed by mean difference (MD) and 95% CI. Statistical heterogeneity analysis will be carried out on the included studies, such as no heterogeneity or small heterogeneity (*I*^2^ ≤ 50%, *P* ≥ .1), and fixed effect model will be used to calculate the amount of combined effect. on the contrary, the heterogeneity is large (*I*^2^ > 50%, *P* < .1), sub group and meta-regression analysis will be used to explore the sources of heterogeneity, including age, sex, disease severity, intervention measures (dosage, dosage form, taste) and treatment course. If there is statistical heterogeneity but no clinical heterogeneity among the research results, the random effect model will be used for meta-analysis.

### 3.9 Publication bias analysis

If there are greater than or equal to 9 studies included in the meta-analysis, we will find publication bias and methodological quality through funnel plot analysis.

### 3.10 Sensitivity analysis

For quality analysis, we will conduct a sensitivity analysis of the main results to explore the influence of the bias of a single study on the results.

## 4 Discussion

RIF is a pathological process in which various primary or secondary renal diseases continue to progress, resulting in the replacement of renal tissue structure by ECM, accompanied by progressive renal function damage. The cytokine-mediated signal transduction pathway plays an important role in this complex process.^[6]^ Therefore, in addition to Scr, BUN and 24-hour urinary protein quantification, which are commonly used in clinical practice to judge renal function, we also select classical fibrosis factors TGF-β1, Notch1 and Jagged1 in TGF-β-Smad signaling pathway and Notch signaling pathway. In the process of RIF, the expression of Notch1 and Jagged-1 is up-regulated in renal tissue, which leads to the increase of serum content. In the absence of fibrosis in other organs except the kidney, the degree of RIF can be explained by the 2.^[16,17]^ Studies confirmed that TGF-β1 is the key cytokine involved in renal tubular fibrosis.^[18,19]^ Measuring the level of TGF-β1 in urine can reflect the net production of TGF-β1. Therefore, the detection of urinary TGF-β1 is of great significance for the determination of RIF.^[20,21]^ TCM has achieved remarkable results in anti-organ fibrosis with its advantages of multi-channel, multi-link and multi-target. The prevention and treatment of RIF with TCM has become a research hotspot in recent years. There have been many reports on RCTs on the treatment of RIF with TCM, but its safety and effectiveness lack corresponding systematic reviews. Therefore, this study will collect relevant clinical studies, systematically analyze and evaluate the therapeutic effect and safety of TCM oral treatment of RIF, and provide scientific reference for its clinical prevention and treatment of TCM. Our research strengths include comprehensive Chinese and English database, rigorous quality assessment and judicious subgroup analysis design. All the above can make our analysis more persuasive. But there are also some limitations in the study, such as the restriction of retrieval language may cause language bias; the severity of the disease, the intervention measures, the course of treatment may have greater clinical heterogeneity, and our future research will pay more attention to these limitations.

## Acknowledgments

The authors would like to thank Yuerong Ma for critically reviewing the manuscript.

## Author contributions

**Data collection:** Guang Yu, Mao Guo.

**Software:** Mao Guo.

**Statistical analysis:** Junju Zou, Xiaotao Zhou.

**Supervision:** Yuerong Ma.

**Writing – original draft:** Guang Yu.

**Writing – review & editing:** Yuerong Ma.

## Abbreviations

Abbreviations: BUN = blood urea nitrogen, CBM = Chinese Biomedical Literature Database, CKD = chronic kidney disease, CNKI = China National Knowledge Infrastructure, Embase = Excerpt Medical Database, ESRD = end-stage renal disease, RCTs = randomized controlled trials, RIF = renal interstitial fibrosis, Scr = serum creatinine concentration, TGF-β1 = transforming growth factor-β1, ECM = extra cellular matrix, VIP = China Science and Technology Journal Database.
